# Exploring Metrics of Biological Diversity to Better Predict and Respond to Emerging Diseases

**DOI:** 10.1093/icb/icaf064

**Published:** 2025-05-29

**Authors:** Andrea Swei, Heather Broughton, Arielle Crews, Marie Lilly, Grace Shaw, Shannon Summers

**Affiliations:** Department of Biology, San Francisco State University, San Francisco, CA 94132, USA; Institute for Integrative Conservation, College of William and Mary, Williamsburg, VA 23185, USA; Department of Biology, San Francisco State University, San Francisco, CA 94132, USA; San Mateo Mosquito and Vector Control District, Burlingame, CA 94010, USA; Department of Ecology, Evolution, and Environmental Biology, Columbia University, New York, NY 10027, USA; Department of Biology, San Francisco State University, San Francisco, CA 94132, USA; Department of Biology, San Francisco State University, San Francisco, CA 94132, USA

## Abstract

The emergence of infectious diseases is largely driven by spillover events from animal communities into human populations, with zoonotic pathogens accounting for 75% of novel infectious agents. In recent years, the incidence and prevalence of these pathogens have been on the rise, and efforts to understand the underlying ecological principles responsible for the reported increases have highlighted the role of biodiversity loss as a major contributing factor. Despite its role in pathogen emergence, how biodiversity is measured can differ drastically and may underlie variability in study results, making the impacts of biodiversity on pathogen behavior difficult to untangle. Here, we first examine how landscape parameters affect disease transmission and then evaluate metrics used in various disease systems to discuss the ways that different aspects of biodiversity, such as functional, phylogenetic, and trophic diversity, can provide novel insight into the relationship between host communities and disease emergence and transmission. We focus on the tick-borne pathogen that causes Lyme disease in this review and discuss how functional, trophic, and phylogenetic diversity can improve our understanding of the relationship between host community structure and disease transmission. The growing public health burden of tick-borne diseases necessitates holistic thinking to inform actions to decrease the risk of disease to humans and protect natural communities.

## Introduction

The emergence of new infectious diseases, as well as the range expansion of existing diseases, begs the questions “why” and “how,” as these events continue to increase around the world (Patz et al. 2000; Jones et al. 2008). There have been many lines of investigation to better understand the drivers of these escalating emergence events. For instance, many studies seek to investigate the relationship between land use patterns (Brownstein et al. 2005; Diuk-Wasser et al. 2021), biodiversity shifts (Keesing et al. 2010; Civitello et al. 2015), or changing environmental conditions (Ogden et al. 2021; Ryan et al. 2021; Baker et al. 2022; Nova et al. 2022) on the transmission dynamics and emergence of pathogens. Results from these studies suggest that the relationship between biodiversity and disease transmission is of particular importance, as over 60% of all emerging zoonotic diseases arise from non-human animals whose pathogens spillover into humans (Jones et al. 2008). Within this context, biodiversity loss driven by anthropogenic disturbances, which includes habitat loss, climate change, and overexploitation, has the ability to change the disease landscape, with significant ramifications for human public health (Ryan et al. 2021; Mora et al. 2022).

**Fig. 1:**
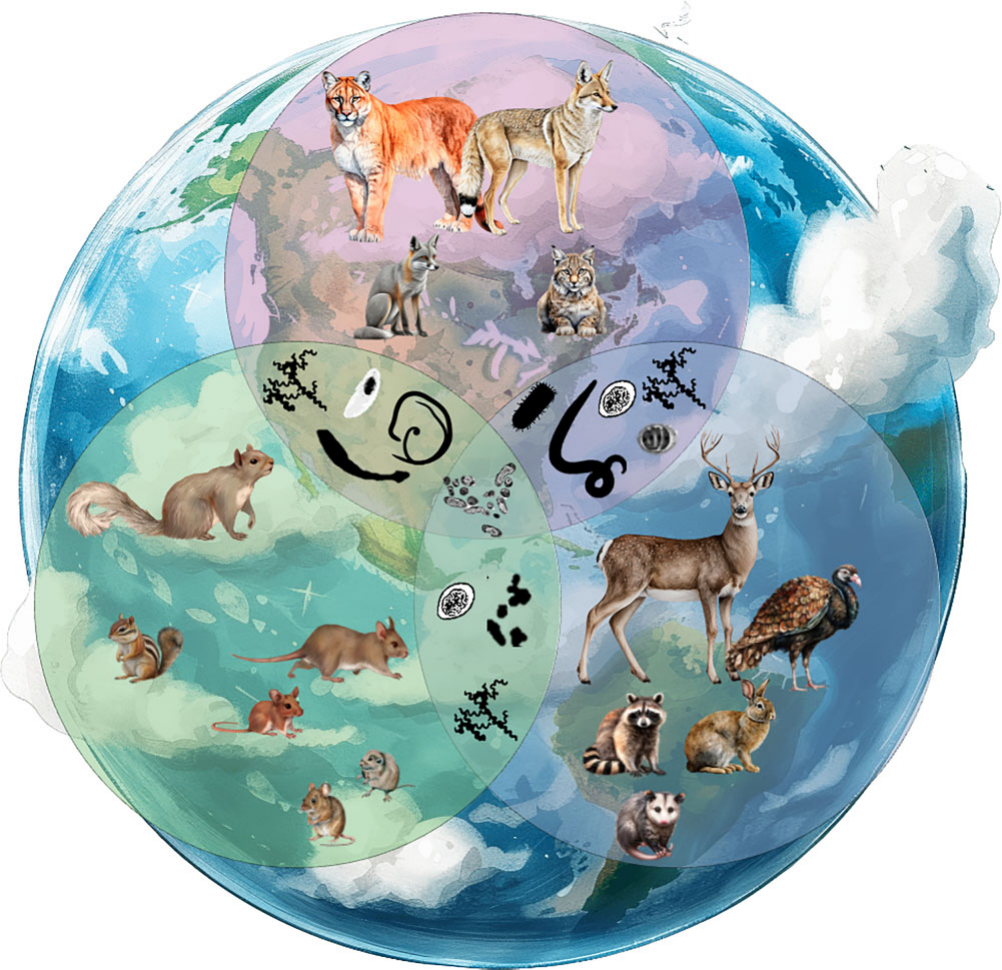
Diagram showing the different aspects of biodiversity that studies may focus on. Rodents in the botton left circle may be the primary reservoirs of a pathogen, but other species may be important competitors (botton right circle) or predators (top circle) that can affect the population dynamics, behavior, and contact rate of focal species. Further, additional species may introduce other pathogens into a community that interact directly or indirectly with the pathogen of interest. Adapted with permission from Light Green Co., 2024; Wall Art Designer, 2024; Nival Color, 2024; Artistic Timber Studio, 2024; Ravenwood Crystal Arts, 2024; Planet Earth 1-10, 2024; and Mona Luo, 2018.

The relationship between biodiversity and pathogen transmission is an important idea in disease ecology that provides a way to understand both “why” and “how” infectious diseases emerge. The idea, termed the dilution effect, states that biodiversity provides an ecosystem service for disease suppression (Ostfeld and Keesing 2000; Schmidt and Ostfeld 2001; Keesing et al. 2010). When this relationship falters, driven mechanistically by biodiversity loss, it is likely to favor species that are better adapted to disturbance and stress. These so-called “fast pace-of-life” species may also be better pathogen reservoirs because their life histories prioritize growth and reproduction over immune defense (Han et al. 2015; Halliday et al. 2020). However, while evidence for the dilution effect has been found in many animal and plant disease systems (Allan et al. 2009; Civitello et al. 2015; Gibb et al. 2020; Halliday et al. 2020; Liu et al. 2020; Magnusson et al. 2020; Keesing and Ostfeld 2021), some studies have found that biodiversity *per se* is not the most important factor, but rather species identity, or the influence of particularly strong interacting species, may be more important for driving observed patterns (Randolph and Dobson 2012; Huang et al. 2014; Wood et al. 2014; Milholland et al. 2017; Wood et al. 2017). Additionally, the metrics used to measure biodiversity vary between studies, with some studies focusing on disease-impacted species while others consider the community as a whole (Fig. 1) (LoGiudice et al. 2008; Allan et al. 2009; Johnson et al. 2015). These variations can make it difficult to draw generalizable conclusions across systems.

The majority of dilution effect studies use host species richness as a measure of biodiversity (Civitello et al. 2015). While this is a relatively easy metric to quantify and can also be accessed across large spatial scales from databases, such as the International Union for Conservation of Nature (International Union for the Conservation of Nature 2024), it can miss important aspects of community composition such as abundance, evenness, and community function. Species abundance, captured in metrics such as Shannon or Simpson's diversity, is labor-intensive to measure and, as a result, is often not included in biodiversity measurements (Kilpatrick et al. 2017; Wood et al. 2017), but there are also many aspects of community composition that are not captured by a simple metric of species richness. For instance, while biodiversity is often linked to the functional diversity of ecosystems, this is not always the case (Giller and O'Donovan 2002; Pennekamp et al. 2018). Species richness also does not distinguish between native and non-native species, the latter of which may respond to habitat fragmentation and biodiversity differently than native species, which may be more tolerant of habitat disturbance and biodiversity loss (Didham et al. 2005). In addition, functional host community diversity and trophic interactions are usually not incorporated into metrics of species richness, but are an important part of understanding biodiverse communities and ecosystem function (Duffy et al. 2007; Thompson et al. 2015).

The field of plant community ecology has long used a variety of approaches to characterize the productivity and ecosystem services provided by ecological communities. Functional, trait, and phylogenetic diversity have all been used to describe plant community diversity and aim to better capture ecosystem productivity and function (Cadotte et al. 2009; Parker et al. 2015; Reemts and Eidson 2019). In this symposium article, we look to this body of plant diversity literature and beyond, to examine how metrics of diversity, many of which were developed across plant and animal community ecology studies, could improve characterizations of biodiversity as well as quantify its functions to help reconcile the variable results reported in the disease ecology literature.

First, we examine the role of landscape structure on both biodiversity and disease risk and then discuss how host functional, trophic, and phylogenetic diversity can be used to more accurately describe the communities that are relevant for understanding pathogen transmission dynamics across different disease systems. Foundational work on the dilution effect was first described in the Lyme disease system in the northeastern United States (Ostfeld and Keesing 2000; Schmidt and Ostfeld 2001; Keesing et al. 2006). Here we look to a different Lyme disease system in a different geographic region that is characterized by a distinct natural history and consider how alternative types of biodiversity metrics could be important and applied. In the western United States, Lyme disease is caused by the bacterium, *Borrelia burgdorferi*, vectored by *Ixodes pacificus* ticks, and maintained by several bloodmeal, reservoir, and reproductive hosts. Some important bloodmeal hosts, like lizards, do not act as pathogen reservoirs, and are actually borreliacidal (Brown and Lane 1992; Lane and Quistad 1998). The inherently community-driven nature of this disease makes it an ideal model system for exploring the role of biodiversity.

### Habitat fragmentation, isolation, and environmental impacts

In some cases, landscape variables such as patch size or habitat type may be sufficient to model the spatial distribution and spread of infectious diseases. This is because, along with abiotic limits, landscape variables are responsible for shaping biodiversity patterns that underlie zoonotic disease dynamics. Since landscape variables and biodiversity are often correlated (Walz 2011; Schindler et al. 2013; Morelli et al. 2018), measuring landscape variables can serve as a simpler and sometimes sufficient proxy for biodiversity. This is especially true if the spatial distribution of a pathogen is large, if the host species in the system is cryptic, or if landscape variables are available while host community data are not. The emergence of tick-borne pathogens, for example, is commonly tracked and predicted using landscape variables such as forest cover, habitat patch fragmentation, and landscape connectivity (Allan et al. 2003; Brownstein et al. 2005; Lambin et al. 2010; Kilpatrick and Randolph 2012; Millins et al. 2018; Diuk-Wasser et al. 2021). Initial efforts to track the emergence of novel infectious diseases may also rely on readily available landscape predictors. For example, landscape features such as urban development were used to predict COVID-19 risk immediately after the emergence of the SARS-CoV-2 virus in Wuhan, China (Ye and Qiu 2021). In an early study tracking the newly emerging tick-borne pathogen, *Rickettsia tillamookensis*, landscape metrics, especially patch distance and isolation, were also the only predictors of pathogen distribution (Shaw et al. 2024).

Though delineating habitat patch boundaries can be difficult and scale-dependent, measuring patch size is generally straightforward (Lyons et al. 2010; Reperant 2010; Spurgin et al. 2012). However, there are numerous metrics available to measure habitat isolation in the context of disease ecology. Choosing an isolation metric requires asking two questions: (1) For the disease system in question, is it more appropriate to measure the simpler metric of isolation distance *or* analyze patch connectivity, which incorporates data about the “matrix” between habitat patches? and (2) In multi-host or vector-borne disease systems, should connectivity be measured through the lens of movement for one (or more) key organisms in the system, or is Euclidean distance a sufficient measurement of isolation? These questions have led to the integration of several connectivity models in the field of disease ecology (Adriaensen et al. 2003; Compton et al. 2007; McRae et al. 2008; McRae and Shah 2009; Lippi et al. 2021; Mai et al. 2024), highlighting the diversity of landscape metrics that can be predictive without the incorporation of biodiversity data.

Careful consideration of the uses of disease models based on environmental parameters provides guidance on when and how to use environmental metrics across infectious disease systems. Key disease systems, such as Lyme disease, have shown that the scale of study is important when choosing between simple metrics of habitat isolation such as Euclidean distance and more sophisticated connectivity analyses such as cost distance, which can incorporate movement data from wildlife communities (Heylen et al. 2019; Shaw et al. 2024). These findings demonstrate that detailed environmental models may predict more accurately at smaller scales, while large-scale study systems can be modeled equivalently with simple metrics that do not take biotic community data into consideration (Shaw et al. 2024). Additionally, landscape modeling approaches have been effective at predicting the spatial risk of wildlife disease via circuit theory connectivity analysis (Nobert et al. 2016), as well as agricultural pest infestations with graph theory (Margosian et al. 2009). These movement studies (Norbert et al. 2016; Heylen et al. 2019; Shaw et al. 2024) present opportunities for model improvement through the use of biotic data, but also show some evidence that simpler models may effectively predict disease distribution even when more complex analyses fall short. These disparate nuances between the pros and cons of including biotic community data hint at the importance of selecting disease system-specific variables in predictive models.

While environmental variables can be both powerful and accessible tools for predicting disease movement and maintenance, they are often limited and may not be able to account for complex disease-specific patterns, including pathogen host preference and differences in host behavior between regions (Ginsberg et al. 2021). When highly detailed and specific landscape data are available, they may overfit the disease model by utilizing landscape variables in the place of more appropriate biotic variables that vary at higher rates between landscapes than their abiotic counterparts (Meier et al. 2010; Williams et al. 2010; Leach et al. 2016). It is important to remember that choosing a landscape metric is inherently scale dependent, as exemplified by the Lyme disease system (Heylen et al. 2019; Shaw et al. 2024). In addition, the impacts of transmission mode, mobility of hosts, free-living stages of a pathogen, and vectors within the system must be considered when utilizing landscape predictors in disease models.

Environmental variables and landscape tools are foundational to the investigation of newly emerging or widespread diseases and should be utilized in the exploratory phases of spatial risk modeling for diseases. In general, they prove more accessible, faster to measure, and more extrapolatable than empirical measurements of biodiversity. Simple landscape metrics may also reduce unnecessary “noise” of complicated, indirect, or mixed relationships between host, vector, and pathogen that are not integral to a specific study or disease control outcome. For large-scale disease studies, landscape metrics can usually be used alone to build an appropriate model. Most importantly, landscape metrics are also integral for predicting diseases unlikely to be influenced by biodiversity, such as host-specific, directly transmitted infections without vectors or free-living stages (Rohr et al. 2020). In many systems, however, it is critical to also directly measure biodiversity to track, predict, and control disease. The addition of biodiversity data in a model specifies the key players in the disease system that can be targeted for disease control and management.

For many zoonotic diseases, especially horizontally transmitted vector-borne systems, incorporation of host community parameters into disease prediction models is necessary because hosts are the reservoirs of the pathogen, some acting as competent hosts while others are dead-end hosts or simply unable to acquire the pathogen from the vector. Measuring biodiversity via host species richness has been the historic first step to more accurate model building in these complex disease systems (Allan et al. 2009; Johnson et al. 2015). In the following sections, we explore more nuanced metrics of biodiversity and each of their roles and applications in the disease modeling field.

### Functional diversity

Functional group diversity, a trait-based categorization of species by their ecological function (Naeem and Wright 2003; Reich et al. 2004; Martin et al. 2019), can be a useful metric to understand the variety of ecological processes or the variety of species that perform overlapping functions within an ecosystem (Naeem and Wright 2003; Ricotta 2005; Blaum et al. 2011; Mammola et al. 2021). This concept has a long legacy in the investigation of plant ecosystem functioning (Hector et al. 1999; Petchey et al. 2005; Pokorny et al. 2005) and plant disease ecology (Garrett et al. 2009), but has been less commonly applied to animal ecology (Blaum et al. 2011). Grouping species into functional groups may be an important conceptual bridge, helping to identify commonalities across plant and zoonotic systems in the ongoing diversity-disease relationship debate.

There are numerous methods for classifying organisms into functional groups. The most common methods include constructing dissimilarity matrices, mathematical models of multidimensional “trait space” (Mammola et al. 2021), or simply focusing on key shared traits of interest for a particular system function (Naeem and Wright 2003). After organisms have been grouped by shared function, there are two dominant ways to consider functional group diversity—either as the diversity *of* functional groups, or diversity *within* functional groups (Mammola et al. 2021). Assessing biological diversity through the lens of diversity *of* functions is most useful when species traits *within* the functional group do not vary drastically in their impact on the process being measured (Reich et al. 2004). For example, in the agricultural sector, the diversity of bee pollination mechanisms may be more important than the diversity of bee pollinator species. In a study of pollinator bee species diversity and crop yield, Hoehn et al. (2008) found that the number of pollinator bee functional groups (grouped by pollination mechanism) was more predictive of crop yield than overall species richness (Hoehn et al. 2008). Examining diversity *within* functional groups, on the other hand, is most useful when the grouping provides information that may be obscured by species richness or diversity alone (Cadotte et al. 2011). In a study of ecosystem stability, Liu et al. (2022) found that richness within fungal functional groups of decomposers compared to plant pathogens had opposite effects on ecosystem stability. Examining overall fungal richness without these functional groupings would conceal the effect of fungal richness within each group on ecosystem functioning (Liu et al. 2022).

In the context of disease ecology, considering the impact of functional group diversity could help target management of host generalist pathogens by identifying specific functions more important to pathogen transmission rather than specific species. For example, Morris et al. (2016) found that *Mycobacterium ulcerans*, a generalist multi-host pathogen that causes the human skin disease Buruli ulcer, was most associated with the presence of host organisms grouped by functional traits related to aquatic feeding preference rather than taxonomic groups (Morris et al. 2016). In a study on potato blight, caused by the pathogen *Phytophthora infestans*, Garrett et al. (2009) found that increased functional divergence within host potato species greatly reduced potato blight disease in potato agricultural systems (Garrett et al. 2009). In a grassland plant disease system, Latz et al. (2012) found that species richness within plant functional groups had differential effects on plant soil-borne pathogen infection and the authors were able to provide grassland ecosystem management suggestions based on their results (Latz et al. 2012).These examples illustrate how functional diversity within a system can be more important in pathogen transmission cycles than species richness or overall diversity.

In the context of Lyme disease, the causative bacterial agent *B. burgdorferi* relies on a multi-host life cycle, making it particularly sensitive to ecological interactions such as competition and predation (LoGiudice et al. 2008; Levi et al. 2012; Ostfeld et al. 2018). Because of the range of host competency and the role they play in both the pathogen and tick host’s life cycle, vertebrate hosts can be grouped by their “function” on pathogen transmission, with each influenced in turn by the community around them. In the western United States Lyme disease system, host community richness *within* functional groups such as rodent reservoirs, non-reservoir bloodmeal hosts, and rodent predators has been found to be more predictive of several metrics of Lyme disease risk than overall vertebrate species richness (Lawrence et al. 2018; Shaw et al. 2024). These findings indicate that overall diversity metrics could obscure important interactions driven by the disease-related functional groups such as the strong influence of rodent diversity on predicting disease risk. Framing communities by functional diversity can offer insights into effects on pathogen transmission dynamics differentially driven by disease-related functional groups.

Functional diversity may be especially useful when there are numerous species with established trait-based, rather than species-specific, roles in pathogen transmission cycles (Naeem and Wright 2003; Reich et al. 2004; Morris et al. 2016; Liu et al. 2022). However, other diversity metrics may be more useful if detailed host community data is unavailable (and therefore diversity within functional groups would be biased), or if traits involved in pathogen transmission are too divergent across species to group them by trait-based functions (Rohr et al. 2020).

### Trophic interactions, trophic diversity, and trophic health

Diseases exist within natural communities that include both the macro community (i.e., ecological community) and infracommunity (i.e., internal parasite assemblage), which interact on all levels to shape host-pathogen and population-level disease dynamics (Keesing et al. 2006; Belden and Harris 2007; Roche et al. 2012; Johnson et al. 2015; Shaw and Civitello 2021). While many of these interspecific interactions can be studied from landscape-level or functional biodiversity perspectives, as is traditional in many sessile plant-based disease systems, biotic interactions between species on non-adjacent trophic levels may also be of profound importance when predicting disease outcomes (Dobson and Hudson 1986; Hatcher and Dunn 2011; Seabloom et al. 2015; Shaw and Civitello 2021; MacDonald et al. 2022). Trophic interactions are roughly defined as indirect interactions between multiple consumer and resource species on non-adjacent trophic levels (i.e., hierarchical levels of the food web; Beckerman et al. 1997; Ripple et al. 2016). While this terminology has been popularized in reference to the impacts of predators on their prey’s resources, namely plants, these interactions can also be applied to the transmission and maintenance of infectious pathogens within an ecological system between indirectly interacting species and hosts (Bowers and Turner 1997; Packer et al. 2003; Rudolf and Antonovics 2005; Keesing et al. 2010; Ripple et al. 2014; Wallach et al. 2015; Buck and Ripple 2017; Frainer et al. 2018; Beschta and Ripple 2020; Su et al. 2022).

Trophic interactions can have positive or negative consequences for a host species, its parasites/pathogens, or other species in its shared community (Belden and Harris 2007). Already, several examples of this have been documented in studies from a range of plant and animal systems. Within the plant literature, which offers the foundation upon which trophic interactions were first studied, examples of trophic-mediated disease dynamics include elevated transmission of pea enation mosaic virus to pea plants by aphids when aphid predators were artificially removed (Clark et al. 2019); protist and symbiont suppression of plant pathogens in the soil microbiome (Guo et al. 2022); and the facilitation of oak species by control of feral and wild swine populations due to outbreaks of African swine fever (Bogdziewicz et al. 2022). Within this context, trophic interactions may take several forms, including disease suppression via predation on a key host, initiation of an extinction vortex due to the combined actions of a predator and a pathogen on a shared host, facilitation of a prey species due to elimination of a key predator by a pathogen, and facilitation of a predator by a pathogen due to death/accessibility to a target prey species, to name a few.

Trophic principles have also been extended to animal systems and show parallel findings. Examples range from gastrointestinal helminth increases in ungulate herds of southern and central Africa due to the extirpation of large carnivores (Packer et al. 2003; Jolles and Ezenwa 2015); disruption of transmission cycles of the parasitic brain worm *Parelaphostrongylus tenuis* by wolf predation in the northern United States and southern Canada (Oliveira-Santos et al. 2021); increased transmission of *Toxoplasma gondii* by domestic cats to rodents, inadvertently causing spikes in toxoplasmosis and encephalitis in non-target species (Richards et al. 2023); as well as the control of poxvirus transmission between squirrels through predation by pine martens (Roberts and Heesterbeek 2021). Numerous studies have also described disease systems where the pathogen itself is responsible for the trophic cascade, such as the economically important pathogen, rinderpest, and its impacts on domestic and free-ranging bovid species in Tanzania (Holdo et al. 2009; Buck and Ripple 2017). Taken together, these mechanisms highlight the need to consider indirect interactions within an ecological context to gain a full accounting of how disease dynamics may play out for the pathogen, its host, or other directly or indirectly interacting sympatric species.

Trophic theory should be considered in the exploration of pathogen dynamics in natural ecosystems, particularly with regards to pathogens exhibiting multi-host life cycles, due to its ability to account for community-driven trends (Wootton et al. 2023). The comparison of multiple direct and indirect species interactions allows exploration of complex trade-offs within multi-species systems, such as those utilized by most vectors, while also allowing more accurate prediction of disease dynamics within an ecological community than pairwise species interactions alone (Duffy et al. 2007; Buck and Ripple 2017).

Tick-borne zoonotic disease systems may be particularly susceptible to trophic mechanisms, as most rely on mid-trophic level reservoir and reproductive hosts (i.e., rodents, herbivores) and require multi-host systems to carry out their life cycle, which are heavily influenced by multiple levels of species interactions (Marcogliese and Cone 1997; Schmidt and Ostfeld 2001; Lafferty et al. 2006; Salkeld and Lane 2010; Hatcher and Dunn 2011; Dunne et al. 2013; Levi et al. 2016; Tsao et al. 2021; Larson et al. 2022). Studies considering only pairwise interactions between pathogen prevalence and reservoir host, or simply viewing species interactions through the lens of overall richness, may thus miss important species interactions that drive disease emergence and maintenance with regards to tick-borne pathogens (Belden and Harris 2007; Levi et al. 2012; Johnson et al. 2015; Levi et al. 2016; Kilpatrick et al. 2017; Terry et al. 2017; Ostfeld et al. 2018; Clark and Crowder 2021; MacDonald et al. 2022). However, despite its potential significance in explaining tick-borne disease dynamics, this area of research remains understudied outside of the plant literature (Clark et al. 2019; Noman et al. 2021; Guo et al. 2022; Santiago et al. 2023) but see Occhibove et al. (2022).

In the western United States Lyme disease system, at least 44 vertebrate species have been recorded in community composition studies (Lawrence et al. 2018; Lilly et al. 2022; Shaw et al. 2024). These species include several known reservoir host species for *B. burgdorferi* (mostly rodent species), reproductive hosts for ticks such as mule deer (*Odocoileus hemionus*), and the non-reservoir juvenile host western fence lizards (*Scleporus occidentalis*) (Brown and Lane 1992; Lawrence et al. 2018; Lilly et al. 2022; Shaw et al. 2024). In addition, several species of meso and apex predators have been recorded, including mountain lions (*Puma concolor*), bobcats (*Lynx rufus*), coyotes (*Canis latrans*), and gray foxes (*Urocyon cinereoargenteus*) (Lilly et al. 2022; Shaw et al. 2024). While the importance of apex and mesopredators for ecosystem health has long been established in community ecology (Packer et al. 2003; Ripple et al. 2014; Wallach et al. 2015; Buck and Ripple 2017), this relationship has rarely been empirically demonstrated. However, preliminary findings in western United States study systems suggest that species such as mountain lions and bobcats likely exert control over *B. burgdorferi* prevalence in their native communities, with marked reductions in prevalence within ticks when these predators are present (Levi and Wilmers 2012; Levi et al. 2012; Salomon et al. 2021). While the mechanisms behind these interactions have yet to be uncovered, it is hypothesized that the presence of predators may affect prey species (e.g., deer and rodents) directly via predation and may also have indirect trophic effects by altering the behavior of intraguild predators and their prey sources (Levi and Wilmers 2012; Levi et al. 2012; Salomon et al. 2021).

However, while trophic theory has significant advantages over other methodologies in its ability to characterize community-wide interactions, there are some difficulties in its application. For one, which species to include and exclude in study design, sampling, and models can be difficult to determine (Wootton et al. 2023). Second, spatial scale can be hard to define due to variability in species behavior and confounding factors such as landscape, climate, and response to human activity, which can pose barriers during analysis of the final dataset if not considered at the outset (Brown et al. 1999; Guzman et al. 2019). Because the parameters of trophic studies are, at their core, defined by the scientists that design them, they can also be heavily influenced by observational bias, with researchers showing a tendency to focus on specific areas of interest or questions (i.e., top-down, bottom-up, evolutionary processes) and thus missing the broader focus (Abdala-Roberts et al. 2019). To avoid these pitfalls, theoretical modelling approaches are recommended at the study's inception to define the boundaries, both spatially and in terms of species interactions, to be included (Zanden and Rasmussen 1999; Baiser and Lockwood 2011; Layman et al. 2012; Terry et al. 2017; Hayden et al. 2021; Liu et al. 2022; Wootton et al. 2023).

Trophic interactions may be particularly important in disease systems where pathogen life cycles rely on multiple hosts or in systems with multiple pathogen reservoirs and long pathogen transmission cycles. Because species interactions are the rule in all ecosystems rather than the exception, focus on single pairwise interactions between species can miss important indirect drivers for disease transmission or maintenance, particularly when a large number of interacting species or extended time periods provide opportunity for outside influence. Therefore, addressing disease concerns through an encompassing species-centered approach, such as trophic theory, may allow better prediction of disease dynamics and outcomes within naturally occurring ecological systems.

### Phylogenetic diversity

Phylogenetic diversity is a measure of biodiversity that explicitly considers the phylogenetic distance between species in a community (Webb et al. 2002). Unlike simpler metrics of biodiversity such as species richness or Shannon diversity, phylogenetic diversity quantifies the evolutionary relatedness of species and may better capture host pathogen competence or susceptibility to infection, which are immunologically and, therefore, evolutionarily determined traits (Webb et al. 2002; Gilbert and Webb 2007; Streicker et al. 2010). This relationship is likely due to the conservation of species’ physiological traits due to more closely related species sharing more similar immunological pathways and mechanisms (Webb et al. 2002; Huang et al. 2013; Olival et al. 2017). Thus, the phylogenetic structure of a wildlife community may have important impacts on disease transmission and risk through host-mediated differences in pathogen or reservoir competency.

Originally described by Faith (1992), phylogenetic diversity was first considered in a plant disease system by Parker et al. (2015), who showed that the intensity of fungal disease was best explained by a plant’s phylogenetic distance to the host species. Since then, this metric has since been used in other plant disease systems (Liu et al. 2016; Gougherty and Davies 2021). For instance, Liu et al. (2016) found a correlation between phylogenetic diversity of plants in a community and the severity of foliar fungal diseases, showing that phylogenetic diversity was the best predictor of disease severity (Liu et al. 2016). While phylogenetic diversity has rarely been extrapolated to animal systems, Wang et al. (2019) accurately predicted livestock pathogen richness and occurrence, showing that the phylogenetic structure of the surrounding wildlife assemblage can shape patterns of livestock diseases in Africa and lending credence to the method as a valuable metric of biodiversity (Wang et al. 2019). However, application of phylogenetic diversity in vector-borne disease systems is rare (Humphreys et al. 2021; Nunn et al. 2021; Wang et al. 2023). Many predictive disease models would benefit from the incorporation of phylogenetic relationships, especially when host contribution to pathogen transmission varies by phylogenetic placement (Fountain-Jones et al. 2018).

Phylogenetic diversity metrics are varied and are calculated from a phylogenetic tree of the community. There are many possible metrics that can be used, each measuring different aspects of the tree and relationships between species. Phylogenetic diversity considers the evolutionary relatedness of species and encapsulates complicated relationships between species in a community in a single metric. These metrics present an opportunity to clarify biodiversity’s role in the dilution effect by specifying which host community compositions truly increase host diversity when it comes to pathogen acquisition and transmission. The three most common are Faith’s phylogenetic diversity, mean pairwise distance, and mean nearest taxon distance (Webb et al. 2002; Tucker et al. 2017; Fountain-Jones et al. 2018).

The greatest limitation on the use of phylogenetic diversity metrics is the lack of genetic data for the vast majority of species, which is necessary for generating accurate phylogenetic trees. Genetic information of species is required to formulate the phylogenetic tree before calculation of these metrics and when this information is lacking researchers must generate their own sequence data, which can be laborious and costly. This is a major drawback to the metric and researchers may choose to concentrate on groups for which phylogenetic data is available or are easier to generate (Karanth et al. 2019). In addition, some phylogenies are debated and can result in multiple tree topographies, leading to subjectivity in tree selection (Suárez-Díaz and Anaya-Muñoz 2008). The choice of metrics can also be daunting for researchers. There are numerous phylogenetic diversity metrics and choosing the correct metric depends on the scope of the study, the research question, and the data available to the researchers. Many resources have been created to help guide researchers on metric choice (Webb et al. 2002; Tucker et al. 2017; Fountain-Jones et al. 2018). Finally, generating phylogenetic diversity metrics is time intensive and requires additional analyses and skills compared to other metrics such as species richness. However, the picante R package (Kembel et al. 2010) has streamlined this process.

There are several mesopredators and an apex predator in the Lyme disease system that may exert strong trophic effects. Complexity of the host community may be captured through the use of these phylogenetic diversity metrics by incorporating host community structure (Parker et al. 2015; Wang et al. 2019). In the Lyme disease system, phylogenetic diversity metrics may be an important indicator of Lyme disease prevalence because they can incorporate the breadth of species genetic diversity in a system where many closely related species (i.e., rodents) play an important part in pathogen transmission, and other species have indirect but still important interactions with pathogen transmission. Emerging evidence suggests that increased phylogenetic diversity is predictive of lower density of infected ticks (Summers et al. *in press*).

Phylogenetic diversity metrics are best used when disease transmission is mediated by the immunogenic responses of host species and there is high variability in host competency, where not all host species have similar responses to the pathogen. If most species in a system exhibit a similar level of reservoir competency, or host range is limited or irrelevant (such as when a pathogen is transmitted via co-feeding [Voordouw 2015]), then a simpler metric may be more appropriate. Incorporating phylogenetic metrics is also useful when the disease system is complex, such as a multi-host or multi-pathogen system where this metric can help improve predictions by incorporating the structure of the host community (Parker et al. 2015; Fountain-Jones et al. 2018). Finally, phylogenetic diversity may be more easily used if a comprehensive phylogenetic tree is already available for the species in question. However, given the tools available to researchers today, this may not be a limiting factor for some disease systems (Karanth et al. 2019).

### Future of disease prevention

Ultimately, the goal of understanding the relationship between biodiversity and disease is to better understand and respond to emerging infectious diseases. Despite efforts to mitigate their spread through preventative techniques such as vaccine and pesticide use, disease cases—such as those of Lyme disease in the United States—continue to rise, highlighting the need for more comprehensive and effective control strategies. Research indicates that disease transmission and emergence are closely linked to host community structure, biodiversity, and habitat changes, emphasizing the importance of incorporating these factors into intervention strategies. Addressing this growing challenge requires an integrated approach that combines ecological understanding, promotes sustainable management practices, and enhances public health communication.

Host-targeted control measures are an increasingly important approach in disease management, offering potential to reduce pathogen transmission in a variety of ecological systems. However, these strategies must be adapted to the specific ecological context to maximize their effectiveness. For instance, in systems where a single reservoir host plays a dominant role in pathogen transmission, targeted vaccines may reduce pathogen prevalence in these hosts. One such example is the use of oral vaccines to manage bovine tuberculosis in badgers, a key wildlife reservoir in the UK and Ireland (Allen et al. 2018). By reducing disease prevalence in badger populations, the risk of spillover to cattle may also be mitigated (Carter et al. 2012; O'Connor and Brunton 2016; Martin et al. 2020; Woodroffe et al. 2024). However, in more complex ecosystems, where multiple reservoir hosts or diverse transmission pathways are involved, more integrated approaches may be necessary. Understanding the role of hosts and community biodiversity as a whole is an important first step to developing a portfolio of mitigation approaches that could include host-targeted vaccines, vector-focused interventions (e.g., acaricides), and habitat management, all tailored to address the unique ecological dynamics of the system.

An additional strategy beyond direct host interventions involves predator-mediated control, a concept that has not yet been empirically tested but may be promising based on the trophic effects of apex predators (Levi et al. 2012; Rohr et al. 2015). As presented above, preserving trophic and functional diversity could offer an ecologically informed approach to managing both human and animal infectious disease. By supporting predator populations—such as through enhancing predator habitat connectivity, prioritizing habitat conservation, and restoring habitat corridors—it may be possible to promote a “One Health” approach to reduce pathogen prevalence (Dantas-Torres et al. 2012; Faburay 2015). This strategy may limit pathogen transmission and promote ecosystem health by enhancing ecosystem function through host genetic diversity and predator/trophic health. When combined with targeted treatments, which identify the species groups most important for disease risk, these integrated strategies may provide a more comprehensive and sustainable approach to controlling tick-borne diseases while fostering ecosystem resilience.

## Conclusion

**Fig. 2:**
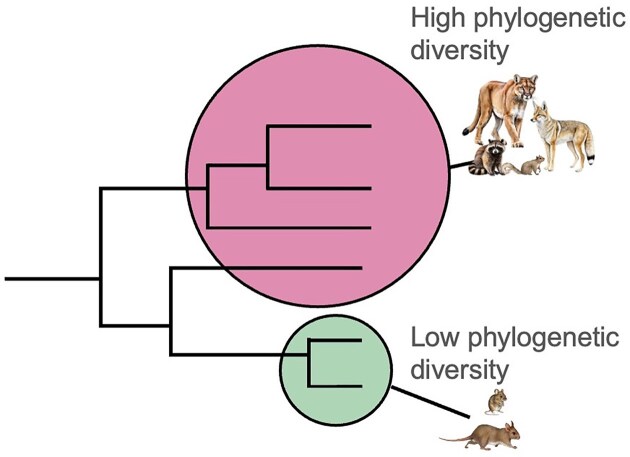
Conceptual illustration of a vertebrate community that has high phylogenetic diversity (composed of carnivores and more distantly related rodents) and low phylogenetic diversity (consisting of only rodents). Adapted with permission from Light Green Company, 2024 and Mona Luo, 2018.

**Fig. 3:**
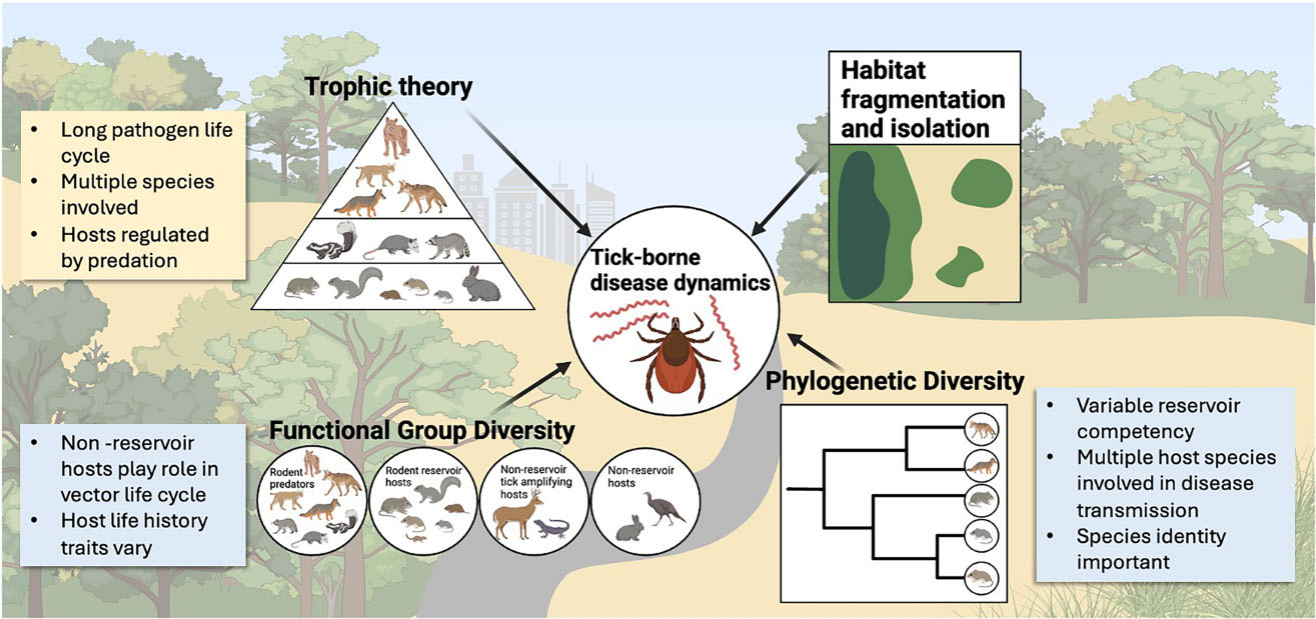
In addition to species richness and landscape variables, analyses evaluating the relationship between biodiversity and disease transmission and risk could also incorporate trophic diversity, functional group diversity, and phylogenetic diversity under certain disease system contexts. Figure created in Biorender. Lilly, M. 2025.

As with many analyses, the choice of metric can have important implications on what patterns are found. While landscape variables or species richness may be appropriate for some simple diseases, systems with long pathogen life cycles where trophic interactions are important may benefit from a trophic theory perspective. Similarly, systems where species identity is particularly strong or where reservoir competency is highly variable, phylogenetic diversity may provide a more accurate measure (Fig. 2). There are circumstances and contexts that may benefit from the use of one metric or another. We propose more deliberate and explicit consideration of biodiversity metrics in disease ecology studies based on the natural history and specific ecology of a given disease system (Fig. 3). Individual disease systems are regulated by different aspects of the host community (overall richness, function/guild group, phylogenetic diversity), and analyses need not be limited to a predictive single metric or response metric (e.g., prevalence or density of infected). Understanding the most important aspects of host community composition paves the way for more ecologically informed disease response and management.

## Author contributions

A.S., H.B., G.S., M.L., A.C., S.S. conceived of the concept of this paper. All authors wrote, edited, and agreed to the final version of the paper.

## Data Availability

Not applicable.

## Notes

Organized symposium “Navigating the Crosscurrents of Plant and Animal Biology to Explore Uncharted Waters in Disease Ecology."

*Brief synopsis:* Using a tick-borne disease system, this review explores how different metrics of biodiversity, as well as how diversity of different functional groups, intersect to shape both pathogen risk and genotypic diversity. In addition, we will examine how these factors shape a broad array of tick-borne pathogen richness.

“From the symposium “Paddling Together: navigating the crosscurrents of plant and animal biology to explore uncharted waters in disease ecology” presented at the annual meeting of the Society for Integrative and Comparative Biology, January 3-7th , 2025.”
